# Changes in serum TIM-3 and complement C3 expression in workers due to Mn exposure

**DOI:** 10.3389/fpubh.2023.1289838

**Published:** 2023-11-09

**Authors:** Yuanmeng Qi, Huifang Si, Xiaofei Jin, Yonghua Guo, Jiarui Xia, Jing He, Xuedan Deng, Meng Deng, Wu Yao, Changfu Hao

**Affiliations:** ^1^Department of Occupational and Environment Health, College of Public Health, Zhengzhou University, Zhengzhou, Henan, China; ^2^Prevention and Infection Control Section, Xi’an Union Hospital, Xi’an, Shanxi, China; ^3^Department of Ultrasound, The Third Affiliated Hospital of Zhengzhou University, Zhengzhou, Henan, China; ^4^Department of Child and Adolescence Health, College of Public Health, Zhengzhou University, Zhengzhou, Henan, China

**Keywords:** Mn-exposed workers, immunotoxicity, RBC Mn, TIM-3, complement C3

## Abstract

Mn (Manganese, Mn) is an essential trace element involved in various biological processes such as the regulation of immune, nervous and digestive system functions. However, excessive Mn exposure can lead to immune damage. Occupational workers in cement and ferroalloy manufacturing and other related industries are exposed to low levels of Mn for a long time. Mn exposure is one of the important occupational hazards, but the research on the effect of Mn on the immune system of the occupational population is not complete, and there is no reliable biomarker. Therefore, this study aimed to evaluate the immunotoxicity of Mn from the soluble immune checkpoint TIM-3 (T-cell immunoglobulin and mucin containing protein 3, TIM-3) and complement C3. A total of 144 Mn-exposed workers were recruited from a bus manufacturing company and a railroad company in Henan Province. An inductively coupled plasma mass spectrometer was used to detect the concentration of RBC Mn (Red blood cell Mn, RBC Mn), and ELISA kits were used to detect serum complement C3 and TIM-3. Finally, the subjects were statistically analyzed by dividing them into low and high Mn groups based on the median RBC Mn concentration. We found that Mn exposure resulted in elevated serum TIM-3 expression and decreased complement C3 expression in workers; that serum TIM-3 and complement C3 expression showed a dose–response relationship with RBC Mn; and that the mediating effect of complement C3 between RBC Mn and TIM-3 was found to be significant. The above findings indicate that this study has a preliminary understanding of the effect of Mn exposure on the immune system of the occupational population exposed to Mn, and complement C3 and TIM-3 may be biomarkers of Mn exposure, which may provide clues for the prevention and control of Mn occupational hazards.

## 1. Introduction

Mn and its compounds are one of the important occupational hazards. Occupational groups such as welders and ferroalloy makers are susceptible to Mn poisoning when exposed at low concentrations for long periods of time (1), and occupational groups are exposed to Mn mainly through inhalation of Mn-containing fumes (2). A large number of studies have shown that Mn exposure has damaging effects on several systems of the body (3), of which the most widely studied is the neurotoxicity of Mn, but the studies on Mn immunotoxicity are not yet comprehensive enough. However, the immune system, as an important system for recognizing and removing harmful substances and maintaining homeostasis of the internal environment, is highly sensitive to toxicity of poisons (4), and the immunotoxic effects of Mn exposure on the organism should not be ignored. When an injury occurs in the organism, changes in the composition of the blood are often its most intuitive manifestation, and it is so sensitive that any stimulus acting on the organism can cause it to change. In epidemiological surveys of populations, changes in certain immune indicators in the peripheral blood of populations can, to a certain extent, well reflect changes in the immune function of the organism after exposure to toxicants.

Blood parameters are used in clinical and toxicologic studies of numerous inflammatory conditions as important physiologic indicators of systemic function (5). When the body is exposed to a toxicant, an inflammatory response occurs, resulting in the formation of a group of cells such as monocytes, T-cells, and platelets, and ongoing inflammation leads to an increase in the number of macrophages and lymphocytes, both of which can migrate from the bloodstream and accumulate at the site of injury. A Finnish study observed the systemic inflammatory response in 20 male workers after acute exposure to welding fumes and found an increase in the total number of blood leukocytes and neutrophils in the workers after shifts (6). Complement C3 is a central component of the complement system and plays a key role in inflammation and regulation of adaptive immune responses (7). Decreased levels of C3 lead to a reduction in the body’s ability to fight infection and a near loss of C3-mediated lysis and clearance of circulating immune complexes. Immune checkpoints are a series of molecules that are expressed primarily on immune cells and regulate the degree of immune activation. Immune checkpoints are capable of generating complex signaling systems that regulate the activation and functional expression of T-lymphocytes, and when they are overexpressed, T-cell immunity is suppressed and tumor cells are able to evade immune killing (8). The immune checkpoint TIM-3, a type I transmembrane protein belonging to the Ig superfamily (9), can be expressed on a wide range of immune cells, including type I helper T cells, Th17 cells, CD8^+^ T cells, and Tregs, and high expression of TIM-3 can trigger immunosuppression as well as tumor immune escape. However, no reports of its expression in studies related to Mn exposure have been found.

It is well known that obesity, smoking and alcohol consumption are common hazards to human health, capable of inducing a wide range of diseases and promoting their occurrence and development. Studies have shown that cigarettes and cigarette smoke contain a variety of toxic trace elements that accelerate damage and inflammation in the human body (10), and smoking directly or indirectly deteriorates blood flow and tissue oxygenation (11). Kim et al. (12) showed that smoking significantly altered the effects of welding fumes on systemic markers of inflammation such as C-reactive protein, fibrinogen and leukocyte levels in the circulation of workers. Several *in vitro* and *in vivo* studies have shown that alcohol modulates the function of innate immune cells such as monocytes and DCs (dendritic cells, DCs) in a dose- and time-dependent manner, and that acute high-dose alcohol consumption suppresses cytokine production, while chronic alcohol consumption stimulates the production of pro-inflammatory cytokines (13). A range of metabolic abnormalities, oxidative stress, immune dysfunction and chronic inflammation have now been identified in overweight obese organisms (14).

Various studies have shown that excessive Mn exposure can affect important organs, leading to multi-system dysfunction. The cumulative evidence of Mn toxicity hazards and the widespread public concern about it fully illustrate its importance to public health. However, the immune system changes induced by occupational Mn exposure in these organs are not fully understood, and the research on the hazards of Mn toxicity in the occupational environment or its nutritional benefits is far from complete. Therefore, the present study conducted a cross-sectional investigation to observe the expression of immune indicators such as WBCs (white blood cells, WBCs), RBCs, Hb (hemoglobin, Hb), PLT (platelets, PLT), TIM-3 and complement C3 in the peripheral blood of Mn exposed workers. Next, the dose–response relationship between the internal exposure dose of RBC Mn and each of the immune markers was analyzed after adjusting for the confounders of working age, smoking, alcohol consumption and BMI. In order to provide clues for the discovery of biomarkers of organismal immune damage caused by occupational Mn exposure, and to provide a reference basis for the prevention and control of occupational Mn exposure toxicity hazards.

## 2. Subjects and methods

### 2.1. Subjects of study

In this study, 144 male Mn-exposed workers, aged 22–53 years old, were recruited from a bus manufacturing company in Henan Province, and their job types were mainly welders. Inclusion criteria: workers aged ≥18 years who had been working in welding or related Mn exposure in the company for more than 1 year, had no other systemic diseases and agreed to participate in the study. Exclusion criteria: those who suffered from significant diseases of the immune system, cardiovascular system, etc. prior to their work related to Mn exposure; those who have recently taken medication that affects their immune function; and workers with a history of other exposures such as benzene and lead. The study complied with medical ethics and was approved by the Life Sciences Ethics Review Committee of Zhengzhou University under the ethical number: ZZUIRB 2022–149. The sample size calculation formula of cross-sectional group design was used in this study:


$$n=\frac{\left({q_{1}}^{-1}+{q_{2}}^{-1}\right){\left(Z_{\alpha /2}+Z_{\beta }\right)}^{2}S^{2}}{\delta^{2}}$$

Sample size: *n*; proportions of two groups: $q_{1}$, $q_{2}$; α = 0.05; test efficacy: 1-*β* = 0.9; standard deviation: *S* = 0.34; tolerable error: *δ* = 0.2. The calculated sample size was 124 people, but 15% was added to account for low cooperation during the survey process, resulting in *n* = 144.

### 2.2. Dust collection and detection

According to the Sampling Specification for Monitoring Hazardous Substances in Air in Workplaces GBZ159-2004, the detection of Mn and its inorganic compounds in the environment of the welding operation is carried out by means of fixed-point sampling, selecting representative workplaces, using AKFC-92A and FC-1B dust samplers with microporous membrane collectors, fixed at the height of the human respiratory belt, with a sampling flow rate of 5 L/min and a sampling time of 15 min, and carrying out sampling in the periods of different concentrations of hazardous substances, including the period of time when the concentration of the hazardous substances in the air is the highest in a working day. At the end of sampling, the microporous filter membrane was folded twice and put into the filter membrane bag. The final compositional analysis was carried out by graphite furnace atomic absorption spectrometer, and the results were determined by the concentration of Mn dioxide, and the lowest detectable concentration of Mn dioxide at the fixed point of this method was 0.006 mg/m^3^ (based on the collection of 75 L of air samples).

### 2.3. Occupational health examinations

Basic demographic information of the study population was collected, as well as basic information such as occupational history, smoking history, and alcohol consumption history. The health checkup includes detection of autonomic symptoms such as headache, palpitation and chest tightness, general physical examination such as height and weight, general clinical examination such as medical-surgical examination and neurological examination, and laboratory biochemical examination such as blood and urine routine and liver function.

### 2.4. Determination of internal exposure dose

Each worker collected 5 mL of biochemical coagulated blood and 2 mL of sodium heparin anticoagulated blood. Blood samples taken on the same day were rested and centrifuged at 1,240 × g for 10 min to retain serum for subsequent detection of soluble immune checkpoints; plasma and blood cells were separated after anticoagulated blood was centrifuged at 600 × g for 5 min. The lower layer of blood cells was washed three times with saline to remove leukocytes, proteins, etc. to obtain red blood cells. Subsequently, RBC Mn content was determined by ICP-MS (Inductively coupled plasma mass spectrometry, ICP-MS).

### 2.5. Measurement of serum TIM-3 and complement C3 expression by enzyme-linked immunosorbent assay

Two replicates were set up for each experimental standard, and serum TIM-3 was assayed using the stock solution uptake assay, and serum was diluted 10,000-fold for the determination of complement C3. A regression equation for the standard curve was calculated using the concentration of the standard versus the OD value, and the sample concentration was calculated by substituting the OD value of the sample into the equation.

### 2.6. Statistical analysis

SPSS 21.0 software was used for statistical analysis. Student’s *t*-test and Mann–Whitney U-test were used to compare the differences in demographic and immunization parameter values between the two groups based on the normality of the distribution of continuous variables. The chi-square test was used for count data. Analysis of covariance and generalized linear models were used to explore the effects of working age, smoking, alcohol consumption and BMI factors on immunological indicators, respectively. Spearman’s correlation coefficient was used to evaluate the correlation between the relevant indicators. After adjusting for working age, smoking, alcohol consumption, and BMI, multiple linear regression and generalized linear models were used to analyze the relationship between internal exposures and each immune parameter. SPSS PROCESS macro was used for mediated effects analyses. The significance level α is 0.05.

## 3. Results

### 3.1. Occupational environment survey

The main source of Mn dust from passenger car manufacturing companies is from welding operations. The raw material used for welding is copper-plated wire, which contains 1.80% ~ 2.10% Mn, and the body frame consists of carbon steel and stainless steel, which can also produce Mn fumes during welding. The ambient monitoring results, as shown in Supplementary Table S1, showed that the 8-h time-weighted average (C-TWA) and peak concentrations of Mn and its inorganic compounds in the air at various points in the workshop were 0.001 ~ 0.142 mg/m^3^ and 0.010 ~ 0.378 mg/m^3^, respectively. The time-weighted average permissible concentration (PC-TWA) for Mn and inorganic compounds (MnO_2_) in our country is 0.15 mg/m^3^, and the peak concentration is three times that of the PC-TWA, 0.45 mg/m^3^.

### 3.2. General demographic features

A total of 144 Mn-exposed male workers were included in this study and their demographic characteristics are described in Table 1. The study subjects were categorized into a low Mn group (*n* = 72) and a high Mn group (*n* = 72) based on the median exposure dose RBC Mn of 2.76 μg/10^10^RBCs. The median age was higher in the low-Mn group than in the high-Mn group, while the overall BMI was greater in the high-Mn group than in the low-Mn group, but none of the differences were statistically significant. The results showed that neither current smoking nor alcohol consumption differed significantly between the two groups; while the distribution of the two groups in different BMIs differed borderline significantly (*p* = 0.087); and the median RBC Mn was higher in the high-Mn group than in the low-Mn group, and the difference was statistically significant (*p* < 0.001).

**Table 1:** Demographic characterization of the study population.

Variables		General population (*n* = 144)	Low Mn group (*n* = 72)	High Mn group (*n* = 72)	*χ*^2^/*Z*	*P*
Age (years)		33 (31, 37)	34 (31, 38)	33 (31, 37)	−0.172*	0.863
	≤33	73 (50.7%)	36 (50.0%)	37 (51.4%)	0.028^#^	0.868
	>33	71 (49.3%)	36 (50.0%)	35 (48.6%)		
Current smoking	Yes	52 (36.1%)	28 (38.9%)	24 (33.3%)	0.482^#^	0.488
	No	92 (63.9%)	44 (61.1%)	48 (66.7%)		
Drinking history	Yes	11 (7.6%)	5 (6.9%)	6 (8.3%)	0.098^#^	0.754
	No	133 (92.4%)	67 (93.1%)	66 (91.7%)		
BMI (kg/m^2^)		24.5 (22.4, 26.9)	24.1 (21.9, 26.2)	25.0 (23.5, 27.1)	−1.584*	0.113
	18.5 ~ 23.9	58 (40.2%)	35 (48.6%)	23 (31.9%)	5.851^#^	0.087
	24.0 ~ 27.9	61 (42.4%)	24 (33.3%)	37 (51.4%)		
	≥28.0	25 (17.4%)	13 (18.1%)	12 (16.7%)		
Working age (years)		8 (5, 10)	8 (6, 11)	8 (5, 10)	−0.815*	0.415
	≤8	82 (56.9%)	41 (56.9%)	41 (56.9%)	0.000^#^	1.000
	>8	62 (43.1%)	31 (43.1%)	31 (43.1%)		
RBC Mn (μg/10^10^ RBCs)		2.76 (1.24, 5.63)	1.26 (0.80, 1.85)	5.63 (3.93, 9.00)	−10.356*	<0.001

*Mann–Whitney U test; ^#^χ^2^-test.

### 3.3. Changes in the expression of peripheral blood immunity indicators in the study population

**Figure 1:**
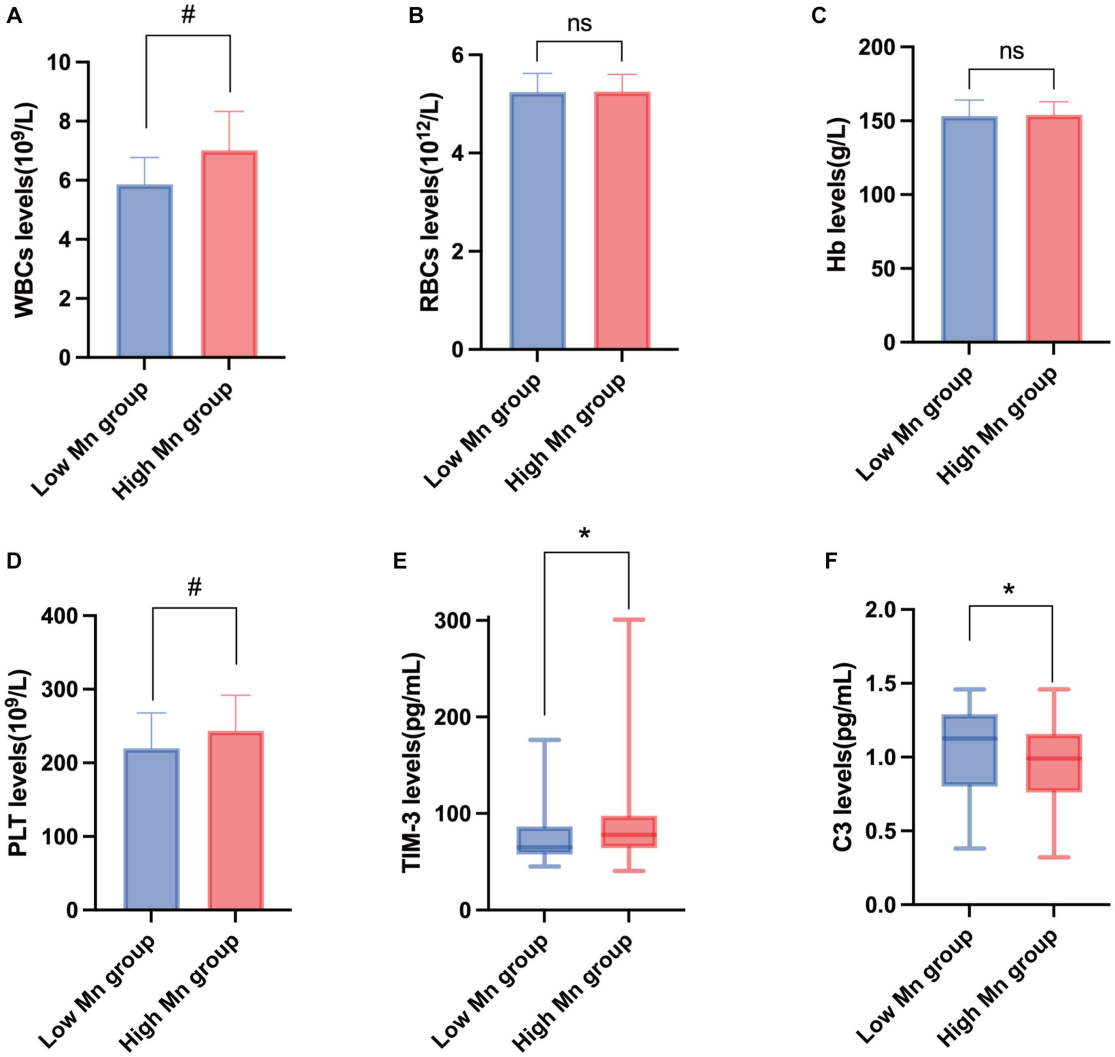
Changes in the expression of peripheral blood immunity indicators in the study population. Changes in the expression levels of WBCs **(A)**, RBCs **(B)**, Hb **(C)**, PLT **(D)**, TIM-3 **(E)**, and C3 **(F)** in peripheral blood of people exposed to manganese. ^#^*T*-test; *Mann–Whitney U test (*n* = 72).

According to the findings shown in Figure 1, a *t*-test analysis revealed that the concentrations of leukocytes and platelets were significantly greater in the high-Mn group compared to the low-Mn group (*p* < 0.001, <0.004, respectively). However, there was no statistically significant difference in the two groups’ RBC and Hb concentrations. To further explore the changes in human immune levels after Mn exposure, workers’ serum TIM-3 and complement C3 levels were measured. We found that TIM-3 was significantly higher in the high-Mn group than in the low-Mn group (*p* < 0.001), with median values of 80.34 and 62.72 pg/mL, respectively. In contrast, complement C3 concentrations were significantly lower in the high Mn group than in the low Mn group, with median values of 0.91 and 1.09 mg/mL, respectively, and the difference between the two groups was significant (*p* = 0.001).

### 3.4. Relationship between demographic characteristics and indicators of peripheral blood immunity

The association of demographic characteristics with WBCs and RBCs is shown in Supplementary Table S2. By intergroup analysis, it was found that in different subgroups of working age, smoking history, drinking history and BMI, the level of WBCs in the high Mn group was higher than that in the low Mn group, and the differences in all subgroups were statistically significant except for the BMI ≥ 28.0 (*p* < 0.05). However, no differences in the expression levels of RBCs were observed between the two groups with different working age, smoking, alcohol consumption and BMI. Within-group analyses showed that in both groups, the levels of WBCs were significantly higher in smokers and drinkers than in non-smokers and non-drinkers, but the difference was statistically significant only in the high-Mn group (*p* < 0.05). In addition, the effect of different BMI on the concentration of WBCs was significant (*p* < 0.05) in the low Mn group, as demonstrated by the relatively high concentration of WBCs in the subjects with high BMI, but not in the high Mn group. On the contrary, the effect of different BMI on the concentration of RBCs was significant (*p* < 0.05) in the per Mn group, and the effect of different working age, smoking and alcohol consumption on the concentration of RBCs was not observed. Supplementary Table S3 shows the relationship between demographic factors and Hb and PLT. Intergroup analysis revealed that PLT levels were higher in the high Mn group than in the low Mn group in the different subgroups of working age, smoking history, drinking history and BMI, where the differences in the different BMI strata were not statistically significant, and the differences between the two groups of those with ≤8 years of working age and those who were smokers were borderline significant (*p* = 0.054, 0.061), and the differences in all the rest of the subgroups were statistically significant (*p* < 0.05). However, no differences in Hb expression levels were observed between the two groups with different working age, smoking, alcohol consumption and BMI. The results of within-group analysis showed that in both groups, the effects of different working age, smoking history, drinking history and BMI conditions on the expression levels of Hb and PLT were not observed, and the differences were not statistically significant (*p* > 0.05). The correlation between demographic variables and the expression of TIM-3 and complement C3 is presented in Supplementary Table S4. Intergroup analysis showed that TIM-3 levels were higher in the high-Mn group than in the low-Mn group for all subgroups except those who drank alcohol, where the differences were not statistically significant (*p* < 0.05). On the contrary, the levels of complement C3 in the high Mn group were observed to be lower than those in the low Mn group in all factor stratifications except for smoking, alcohol consumption, and obesity, and the difference was statistically significant (*p* < 0.05). Within-group analysis revealed that in the low-Mn group, complement C3 levels were higher in those with >8 years of working age than in those with ≤8 years of working age, with a statistically significant difference (*p* < 0.05), and lower in those who smoked and drank alcohol than in those who did not, with a statistically significant difference in both cases (*p* < 0.05). The effects of different working age, smoking, alcohol consumption and BMI status on TIM-3 concentration were not observed.

### 3.5. Relationship between internal exposure dose and peripheral blood immunity indices

**Figure 2:**
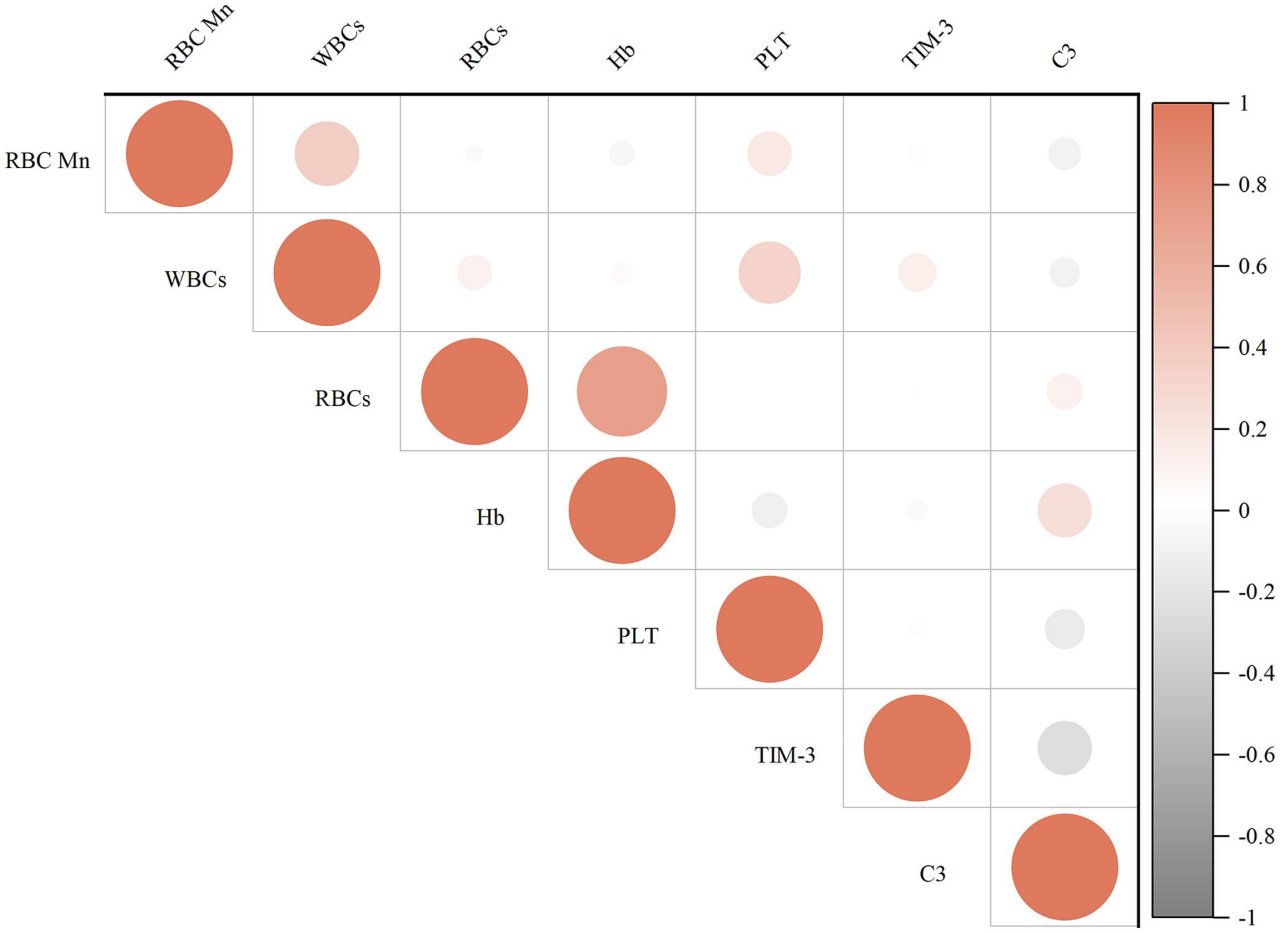
Correlation analysis between indicators.

**Figure 3:**
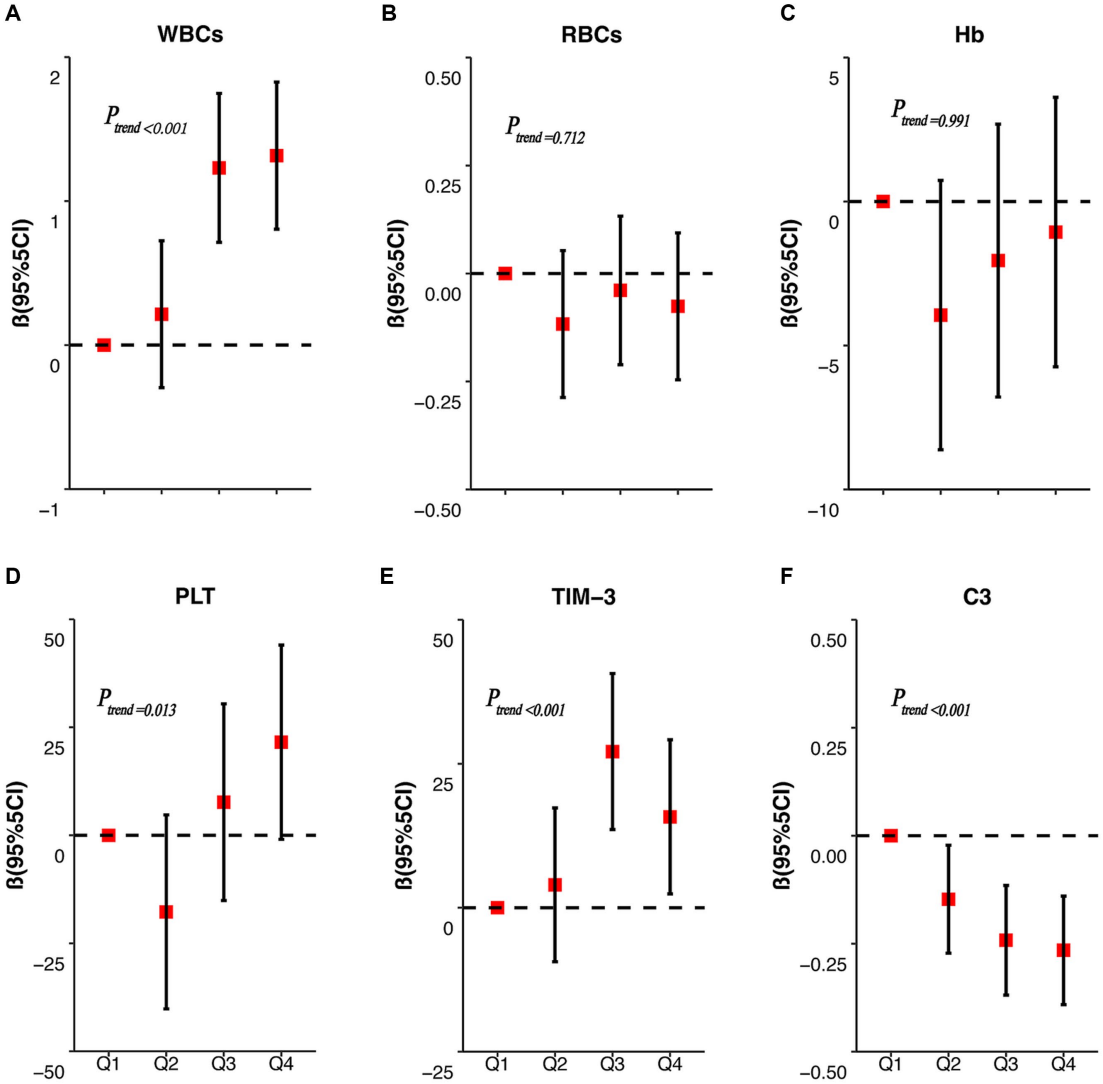
RBC Mn dose–response correlations with WBCs, RBCs, Hb, PLT, TIM-3, and complement C3. The dose–response relationships between RBC Mn concentration and peripheral blood WBCs **(A)**, RBCs **(B)**, Hb **(C)**, PLT **(D)**, TIM-3 **(E)** and C3 **(F)** concentrations were analyzed.

Figure 2 displays the results of the correlation analysis between the internal exposure dose and each peripheral blood immunity index. Spearman’s correlation test revealed that WBCs (*r* = 0.385), PLT (*r* = 0.190), and TIM-3 (*r* = 0.338) were positively correlated with the concentration of RBC Mn (*p* < 0.05); whereas complement C3 (*r* = −0.302) was negatively correlated with the concentration of RBCMn (*p* < 0.01); Hb (*r* = 0.766) was positively correlated with the concentration of RBCs, PLT (*r* = 0.259) and WBCs were positively correlated (*p* < 0.01); complement C3 (*r* = 0.204) was positively correlated with Hb (*p* < 0.05); and complement C3 (*r* = −0.343) was negatively correlated with TIM-3 (*p* < 0.01). We then evaluated the dose–response relationship between erythrocyte Mn levels and peripheral blood immunity indices. The participants in the study were categorized into four groups based on the quartiles of RBC Mn. Subsequently, the researchers examined the association between RBC Mn levels and various indices of peripheral blood immunity. To account for potential confounding factors such as working age, history of smoking, history of alcohol consumption, and BMI, appropriate adjustments were made. The results of this analysis can be found in Figure 3. The findings of the study indicate that there was a significant positive association between the concentrations of WBCs, PLT, and TIM-3 with increasing RBC Mn concentration (*P*_trend_ < 0.001, 0.013, and < 0.001, respectively). Conversely, there was a significant negative association between the concentration of complement C3 and increasing RBC Mn concentration (*P*_trend_ < 0.001). Furthermore, no significant dose–response relationship was observed between the concentrations of RBCs, Hb, and RBC Mn. Compared with RBC Mn < 1.24 μg/10^10^ RBC, both WBCs and TIM-3 concentrations were significantly higher when RBC Mn ≥ 2.76 μg/10^10^ RBC (*p* < 0.05), and the differences between other levels were not statistically significant. In addition, compared with RBC Mn < 1.24 μg/10^10^ RBC, complement C3 concentrations were significantly lower at all other RBC Mn concentration levels, and the differences were statistically significant (*p* < 0.05).

### 3.6. Mediating effects of complement C3 between RBC Mn and TIM-3

**Figure 4:**
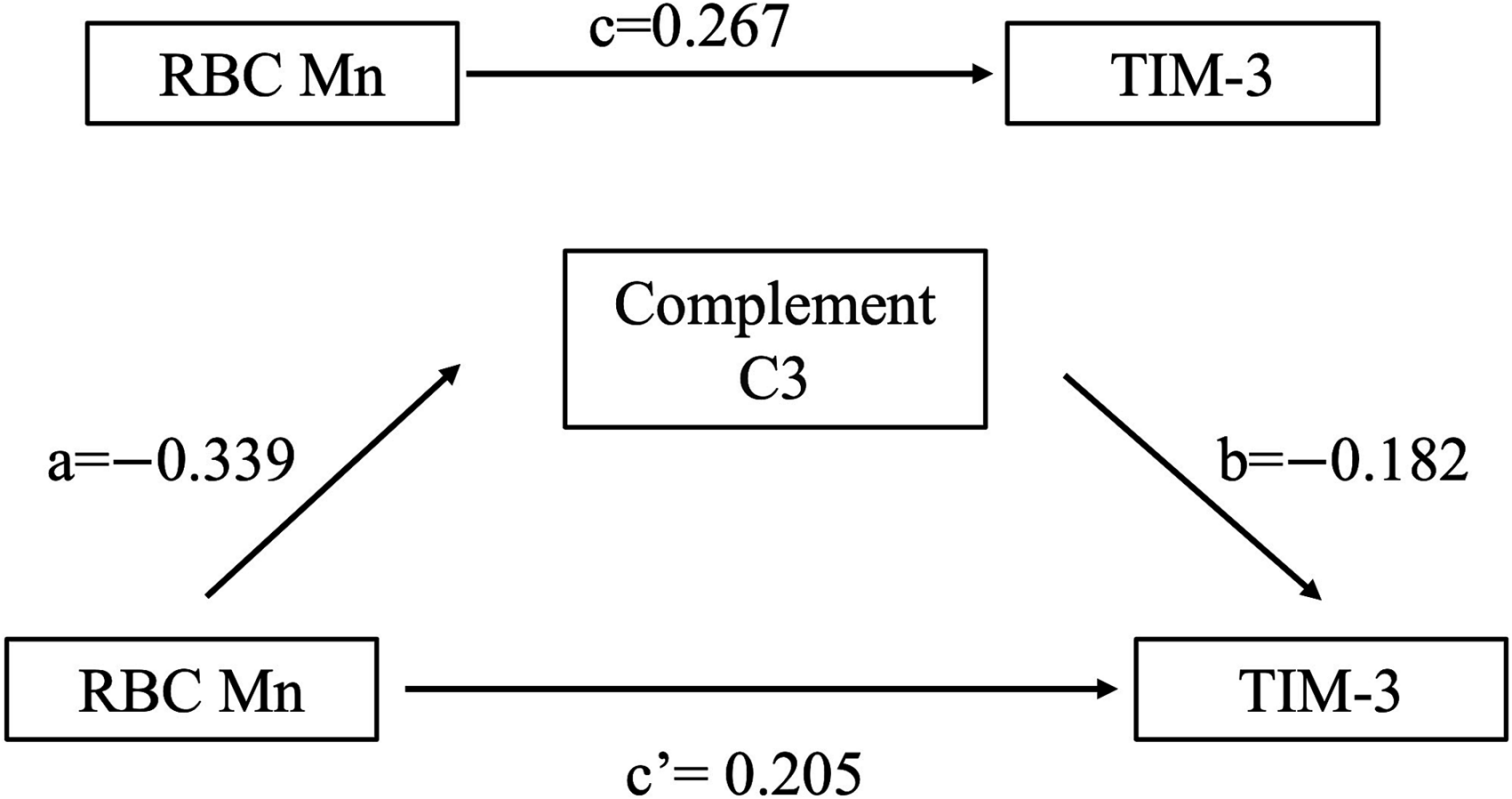
Analytical model of the mediating effect of complement C3 between RBC Mn and TIM-3.

First, the hypothesis was established that complement C3 mediates the effect between RBC Mn exposure levels and changes in TIM3 expression levels, as shown in Figure 4. The PROCESS macro of SPSS was used for mediation effect analysis and the results, as indicated in Supplementary Table S5. The mediating effect was found to be significant for the direct effect of RBC Mn on altered peripheral blood TIM-3 expression levels (*B* = 0.267, *p* = 0.01). After the addition of the intermediate variable complement C3 between RBC Mn and TIM-3, the B of RBC Mn and TIM-3 decreased from 0.267 to 0.205, but remained significant (*p* = 0.0034). The mediating effect was 0.062, and the percentage of mediating effect was 23.2%, demonstrating that the idea that complement C3 partially mediates the interaction between RBC Mn and TIM-3 is correct.

## 4. Discussion

Currently, in welding, ferroalloy manufacturing, and other related industries, workers are in long-term low-level exposure environments where occupational populations are exposed to Mn primarily through inhalation and dermal contact (15). Studies have shown that Mn exposure can lead to immune damage, and long-term inhalation of Mn-containing fumes can even cause chronic Mn poisoning. Occupational Mn poisoning is one of the legally recognized occupational diseases in China, showing irreversible pathological changes in the late stage, and there is no sensitive diagnostic criterion yet. Therefore, strengthening the assessment and monitoring of the working environment of the relevant occupational groups and actively exploring reliable biomarkers are urgent issues that we need to address.

In recent years, an increasing number of studies have focused on the changes in blood parameters after exposure of the organism to Mn in order to evaluate the toxic effects of Mn. The results of this study showed a significant increase in the number of WBCs and PLTs in the peripheral blood of workers in the high Mn group relative to the low Mn group, but the changes in the number of erythrocytes and hemoglobin were not significant, which is consistent with the results of most studies (16, 17). Chen et al. (18) found that occupational Mn exposure had a significant immunotoxic effect on workers, wherein workers in the high Mn exposure group had increased blood WBCs concentrations relative to those in the low Mn exposure group, although not statistically significant. The above results were also verified in animal experiments, after Mn treatment in acute and subchronic model rats constructed with MnCl_2_ (manganese chloride, MnCl_2_), PLTs and WBCs were significantly elevated in the Mn dyed group, in which the number of neutrophils and eosinophils was significantly increased and the number of lymphocytes was decreased (16, 19). More interestingly, the above results were also observed in rats perfused with Mn-containing welding fumes and in fish exposed to Mn (20–22). Taken together, Mn exposure caused an increase in the number of leukocytes, probably due to an increase in neutrophils, which are the main inflammatory cells. Platelets, as one of the indicators of blood inflammation, not only play a hemostatic role, but also produce IgG (Immunoglobulin G, IgG) to play an immune role, while Mn exposure can promote the formation of reactive oxygen species and the consumption of antioxidant elements in the body, which in turn induces the activation of platelets in the body and contributes to the increase of platelet production.

With the discovery of aberrant expression of immune checkpoint molecules in a variety of tumors and autoimmune diseases, there is increasing evidence that they play an important role in maintaining homeostasis within the body’s immune system. TIM-3 is an inhibitory immune checkpoint receptor structurally expressed on activated T cells that protects healthy cells from excessive inflammatory or autoimmune responses by binding to its ligand Gal-9 (23). Soluble TIM-3 is a form of its presence in the blood that is both responsive to immune changes and easily accessible, making it suitable as an effector marker. At present, domestic and international studies on Mn and immune checkpoints are mainly focused on the field of Mn^2+^ as an interferon gene-stimulating factor agonist combined with immune checkpoint inhibitors for antitumor therapy (24), but no studies on immune checkpoints as markers of Mn exposure effects have been found. Considerably, several studies have demonstrated that high concentrations of TIM-3 can be detected in plasma or serum of cancers such as lung, gastric, liver, and cervical cancers and systemic inflammatory patients such as those with chronic hepatitis B virus infection and transplantation of patients with acute leukemia (25), suggesting that it may be associated with a poor prognosis of the disease, affirming its value as a prognostic or predictive marker for predicting the disease or assessing the response to immunotherapy. In this study, after controlling for smoking, alcohol consumption and obesity influencing factors, the dose–response relationship between Mn internal exposure dose and TIM-3 was analyzed and it was found that the concentration of TIM-3 in the serum of the workers tended to increase with the increase of the internal exposure dose, which indicated that the measured circulating level of TIM-3 may be correlated with the dose of the toxic exposure, suggesting that TIM-3 has a potential value as a marker of the effect of Mn exposure. Complement C3 is a core component of the complement system and plays a crucial role in defense against pathogens, removal of apoptotic cells, enhancement of phagocytosis, inflammation, and regulation of adaptive immune responses (26). Chen et al. administered different doses of MnSO_4_-H_2_O to SD rats by intraperitoneal injection and found that Mn staining showed a biphasic dose–response relationship with complement C3, exhibiting low-dose stimulatory and high-dose inhibitory effects (18). However, it has also been found in animal experiments that the effect of Mn-treated welding fumes on the concentration of complement C3 in the rat circulation was not significant when compared to the control group (20). In population-based epidemiologic surveys, it has been found that male workers in the high Mn-exposed group had slightly lower levels of complement C3, which is consistent with the results of the present study. The results showed that the levels of complement C3 in exposed workers tended to decrease significantly with increasing RBCs Mn content, but the reason for this is not clear. The hepatotoxicity of Mn has been demonstrated in several studies (27, 28), but whether chronic repeated exposure to Mn impairs liver function and thus leads to a decrease in complement C3 values in humans remains to be investigated.

The subjects included in this study were all male welders and there was no difference in working age, smoking and alcohol consumption between the low and high Mn groups, with only a borderline significant difference in the subgroups with different BMIs (*p* = 0.087). Fatma Ates Alkan et al. (29) demonstrated that smoking not only altered hematological indices such as whole blood and plasma viscosity, fibrinogen, WBCs and RBCs, but also Mn trace element levels in smokers. At the same time, elevated serum Mn levels in the body aggravate tissue oxygenation, which in turn leads to disturbances in defense mechanisms and respiratory dysfunction. This study found that smokers had higher leukocyte and platelet concentrations and lower complement C3 concentrations in the high Mn group, suggesting that smoking worsens Mn′s immune system effects. Alcohol consumption is known to affect both cellular and humoral immunity (30, 31), as evidenced by a decrease in the number of lymphocytes, as well as an increase in the number of immunoglobulins. In contrast, the results of the present study showed that in the high Mn group drinkers had significantly higher leukocyte counts than non-drinkers, whereas in the low Mn group drinkers had lower levels of complement C3 than non-drinkers, but the statistical difference was borderline significant (*p* = 0.098). The increase in the number of leukocytes in this case may be due to the fact that alcohol consumption exacerbates the inflammatory effect of Mn on the body, leading to an increase in its main inflammatory cells, the neutrophils, the exact mechanism of which needs to be further investigated. Obesity is now recognized as an important risk factor for cardiovascular disease, immune disorders, and many types of cancer. According to the findings of this study, leukocyte, erythrocyte, and platelet concentrations in the peripheral blood of exposed employees rose with increasing BMI, demonstrating that obesity is a risk factor for Mn exposure. Among them, in the low Mn group, there were significant differences in the number of leukocytes between different BMIs; in the high Mn group, there were significant differences in the number of erythrocytes between different BMIs. Obese individuals have increased metabolism and oxygen consumption in their own tissues and organs due to fat accumulation and excessive weight gain; however, obese individuals tend to be in a state of chronic relative hypoxia, and hypoxia stimulates increased secretion of erythropoietin by renal proximal glomerulonephritis (32), which in turn enhances the function of the bone marrow hematopoietic system, leading to increased erythropoiesis.

The complement system plays a predominantly inhibitory role in T cell-mediated antitumor immunity, and multiple immune checkpoints activated on T cells have been identified and applied to the treatment of tumors and immune-related diseases. In recent years, the use of the complement system in immune checkpoint therapy has received increasing attention from researchers. Many components of the complement system can be involved in tumor immune escape mechanisms by down-regulating T cell activity and immune response through complement-dependent or auto-mechanisms. Understanding the role that complement plays in targeting immune checkpoint proteins is of paramount importance, and may improve therapeutic efficacy as well as reduce drug resistance. One investigator demonstrated that upregulation of checkpoint ligand programmed death ligand 1 in patients with paroxysmal nocturnal hemoglobinuria was explained by proximal complement activation (33). Shao et al. (34) investigators found that inhibition of EGFR up-regulated CD55 and CD59 expression activated the complement system and made lung cancer sensitive to checkpoint blockade. The results of the current study showed that there was a partial mediating effect of complement C3 in the process of RBC Mn affecting the changes in the expression level of TIM-3 at immune checkpoints, and the mediating effect accounted for 23.2%, which suggests that complement C3 is able to modulate the effect of RBC Mn exposure on the changes in the expression level of TIM-3 at immune checkpoints. Therefore, further clarification of the mechanism of interaction between complement C3 and the immune checkpoint TIM-3 may provide a new research strategy for occupational hazards of Mn exposure.

## 5. Conclusion

In conclusion, the present study showed that Mn exposure could increase the expression of TIM-3 and decrease the expression of complement C3 in the serum of workers, suggesting that Mn has an inhibitory effect on the body’s immune system. Smoking, drinking and high BMI can also affect the expression of peripheral blood immune indicators. There is a dose–response relationship between the expression levels of TIM-3 and complement C3 in serum and the level of Mn in red blood cells in workers exposed to Mn, and complement C3 has a significant mediating effect between Mn and TIM-3 in red blood cells, which can provide clues for the discovery of biomarkers of Mn exposure. This study can preliminarily understand the effect of Mn exposure on the body’s immune system, and lay the foundation for the study of biomarkers of Mn exposure. However, the research is not comprehensive, and there is no in-depth study on the specific mechanism of changes in the immune system caused by Mn exposure, which needs to be further improved in the future to provide a better basis for the prevention and treatment of Mn immunotoxicity. Therefore, in the future, we will use an animal model of Mn exposure to further explore the immunotoxic effect of Mn, focusing on the changes in histopathology, T cell count, immunoglobulin, related immune checkpoints and cytokines in rats after Mn exposure. To identify the key biomarkers of Mn exposure, use targeted intervention technologies such as small molecule inhibitors and gene editing to intervene the key biomarkers in the Mn exposure model, comprehensively observe the intervention effect, and evaluate the targeted intervention value of related biomarkers.

## Data availability statement

The raw data supporting the conclusions of this article will be made available by the authors, without undue reservation.

## Ethics statement

The studies involving humans were approved by the Life Science Ethics Review Committee of Zhengzhou University/Zhengzhou University. The studies were conducted in accordance with the local legislation and institutional requirements. The ethics committee/institutional review board waived the requirement of written informed consent for participation from the participants or the participants’ legal guardians/next of kin because We verbally informed the study subjects of the purpose of the survey and obtained their consent.

## Author contributions

YQ: Data curation, Investigation, Writing – original draft. HS: Data curation, Formal analysis, Writing – review & editing. XJ: Validation, Writing – original draft. YG: Supervision, Writing – review & editing. JX: Data curation, Writing – review & editing. JH: Validation, Writing – review & editing. XD: Investigation, Writing – review & editing. MD: Investigation, Writing – review & editing. WY: Conceptualization, Supervision, Writing – review & editing. CH: Supervision, Writing – review & editing.

## References

[1] Racette BA Aschner M Guilarte TR Dydak U Criswell SR Zheng W. Pathophysiology of Mn-associated neurotoxicity. Neurotoxicology. (2012) 33:881–6. doi: 10.1016/j.neuro.2011.12.010, PMID: 22202748 PMC3350837

[2] Jiang YM Mo XA du FQ Fu X Zhu XY Gao HY. Effective treatment of Mn-induced occupational parkinsonism with p-aminosalicylic acid: a case of 17-year follow-up study. J Occup Environ Med. (2006) 48:644–9. doi: 10.1097/01.jom.0000204114.01893.3e, PMID: 16766929 PMC4180660

[3] O’Neal SL Zheng W. Mn toxicity upon overexposure: a decade in review. Curr Environ Health Rep. (2015) 2:315–28. doi: 10.1007/s40572-015-0056-x, PMID: 26231508 PMC4545267

[4] Wang Z Sun Y Yao W Ba Q Wang H. Effects of cadmium exposure on the immune system and Immunoregulation. Front Immunol. (2021) 12:695484. doi: 10.3389/fimmu.2021.695484, PMID: 34354707 PMC8330548

[5] Li H Hedmer M Kåredal M Björk J Stockfelt L Tinnerberg H. A cross-sectional study of the cardiovascular effects of welding fumes. PLoS One. (2015) 10:e0131648. doi: 10.1371/journal.pone.0131648, PMID: 26147298 PMC4492943

[6] Järvelä M Kauppi P Tuomi T Luukkonen R Lindholm H Nieminen R. Inflammatory response to acute exposure to welding fumes during the working day. Int J Occup Med Environ Health. (2013) 26:220–9. doi: 10.2478/s13382-013-0097-z, PMID: 23690265

[7] Mathern DR Heeger PS. Molecules great and small: the complement system. Clin J Am Soc Nephrol. (2015) 10:1636–50. doi: 10.2215/CJN.06230614, PMID: 25568220 PMC4559511

[8] Zhang Y Zheng J. Functions of immune checkpoint molecules beyond immune evasion. Adv Exp Med Biol. (2020) 1248:201–26. doi: 10.1007/978-981-15-3266-5_9, PMID: 32185712

[9] Freeman GJ Casasnovas JM Umetsu DT DeKruyff RH. TIM genes: a family of cell surface phosphatidylserine receptors that regulate innate and adaptive immunity. Immunol Rev. (2010) 235:172–89. doi: 10.1111/j.0105-2896.2010.00903.x, PMID: 20536563 PMC2914464

[10] Lee J Taneja V Vassallo R. Cigarette smoking and inflammation: cellular and molecular mechanisms. J Dent Res. (2012) 91:142–9. doi: 10.1177/0022034511421200, PMID: 21876032 PMC3261116

[11] Northrop-Clewes CA Thurnham DI. Monitoring micronutrients in cigarette smokers. Clin Chim Acta. (2007) 377:14–38. doi: 10.1016/j.cca.2006.08.028, PMID: 17045981

[12] Kim JY Chen JC Boyce PD Christiani DC. Exposure to welding fumes is associated with acute systemic inflammatory responses. Occup Environ Med. (2005) 62:157–63. doi: 10.1136/oem.2004.014795, PMID: 15723880 PMC1740976

[13] Ceni E Mello T Galli A. Pathogenesis of alcoholic liver disease: role of oxidative metabolism. World J Gastroenterol. (2014) 20:17756–72. doi: 10.3748/wjg.v20.i47.17756, PMID: 25548474 PMC4273126

[14] Pararasa C Bailey CJ Griffiths HR. Ageing, adipose tissue, fatty acids and inflammation. Biogerontology. (2015) 16:235–48. doi: 10.1007/s10522-014-9536-x 25367746

[15] Sjögren B Albin M Broberg K Gustavsson P Tinnerberg H Johanson G. An occupational exposure limit for welding fumes is urgently needed. Scand J Work Environ Health. (2022) 48:1–3. doi: 10.5271/sjweh.4002, PMID: 34821369 PMC8729164

[16] Ismail HTH. Hematobiochemical disturbances and oxidative stress after subacute Mn chloride exposure and potential protective effects of Ebselen in rats. Biol Trace Elem Res. (2019) 187:452–63. doi: 10.1007/s12011-018-1395-x 29858966

[17] Scharrer E Hessel H Kronseder A Guth W Rolinski B Jörres RA. Heart rate variability, hemostatic and acute inflammatory blood parameters in healthy adults after short-term exposure to welding fume. Int Arch Occup Environ Health. (2007) 80:265–72. doi: 10.1007/s00420-006-0127-2, PMID: 16791613

[18] Chen X Liu Z Ge X Luo X Huang S Zhou Y. Associations between Mn exposure and multiple immunological parameters in Mn-exposed workers healthy cohort. J Trace Elem Med Biol. (2020) 59:126454. doi: 10.1016/j.jtemb.2020.126454, PMID: 31954213

[19] Bahar E Lee GH Bhattarai KR Lee HY Kim HK Handigund M. Protective role of quercetin against Mn-induced injury in the liver, kidney, and lung; and hematological parameters in acute and subchronic rat models. Drug Des Devel Ther. (2017) 11:2605–19. doi: 10.2147/DDDT.S143875, PMID: 28919711 PMC5592961

[20] Antonini JM Zeidler-Erdely PC Young SH Roberts JR Erdely A. Systemic immune cell response in rats after pulmonary exposure to Mn-containing particles collected from welding aerosols. J Immunotoxicol. (2012) 9:184–92. doi: 10.3109/1547691X.2011.650733, PMID: 22369286

[21] Aliko V Qirjo M Sula E Morina V Faggio C. Antioxidant defense system, immune response and erythron profile modulation in gold fish, *Carassius auratus*, after acute Mn treatment. Fish Shellfish Immunol. (2018) 76:101–9. doi: 10.1016/j.fsi.2018.02.042, PMID: 29481848

[22] Sani A Abdullahi IL. Effects of welding fumes on haematological parameters of male albino rats (*Rattus norvegicus*). Biochem Biophys Rep. (2019) 19:100651. doi: 10.1016/j.bbrep.2019.100651, PMID: 31289757 PMC6593231

[23] Tang R Rangachari M Kuchroo VK. Tim-3: a co-receptor with diverse roles in T cell exhaustion and tolerance. Semin Immunol. (2019) 42:101302. doi: 10.1016/j.smim.2019.101302, PMID: 31604535

[24] Lv M Chen M Zhang R Zhang W Wang C Zhang Y. Mn is critical for antitumor immune responses via cGAS-STING and improves the efficacy of clinical immunotherapy. Cell Res. (2020) 30:966–79. doi: 10.1038/s41422-020-00395-4, PMID: 32839553 PMC7785004

[25] Yegin ZA Can F Aydın Kaynar L Gökçen S Eren Sadioğlu R Özkurt ZN. Pre-transplant sTIM-3 levels may have a predictive impact on transplant outcome in acute leukemia patients. Hematology. (2020) 25:125–33. doi: 10.1080/16078454.2020.1738097, PMID: 32153257

[26] del Tordello E Vacca I Ram S Rappuoli R Serruto D. *Neisseria meningitidis* NalP cleaves human complement C3, facilitating degradation of C3b and survival in human serum. Proc Natl Acad Sci U S A. (2014) 111:427–32. doi: 10.1073/pnas.1321556111, PMID: 24367091 PMC3890809

[27] Fordahl S Cooney P Qiu Y Xie G Jia W Erikson KM. Waterborne Mn exposure alters plasma, brain, and liver metabolites accompanied by changes in stereotypic behaviors. Neurotoxicol Teratol. (2012) 34:27–36. doi: 10.1016/j.ntt.2011.10.003, PMID: 22056924 PMC3268843

[28] Huang P Chen C Wang H Li G Jing H Han Y. Mn effects in the liver following subacute or subchronic Mn chloride exposure in rats. Ecotoxicol Environ Saf. (2011) 74:615–22. doi: 10.1016/j.ecoenv.2010.08.011, PMID: 20813406

[29] Ates Alkan F Karis D Cakmak G Ercan AM. Analysis of the relationship between Hemorheologic parameters, aluminum, Mn, and selenium in smokers. Biol Trace Elem Res. (2019) 187:22–31. doi: 10.1007/s12011-018-1352-8, PMID: 29704205

[30] Bhatia R Thompson C Ganguly K Singh S Batra SK Kumar S. Alcohol and smoking mediated modulations in adaptive immunity in pancreatitis. Cells. (2020) 9:1880. doi: 10.3390/cells9081880, PMID: 32796685 PMC7463831

[31] Gonzalez-Quintela A Alende R Gude F Campos J Rey J Meijide LM. Serum levels of immunoglobulins (IgG, IgA, IgM) in a general adult population and their relationship with alcohol consumption, smoking and common metabolic abnormalities. Clin Exp Immunol. (2008) 151:42–50. doi: 10.1111/j.1365-2249.2007.03545.x, PMID: 18005364 PMC2276914

[32] Wakabayashi I. Associations between polycythemia and cardiometabolic risk factors in middle-aged men. Clin Chim Acta. (2022) 531:248–53. doi: 10.1016/j.cca.2022.04.009, PMID: 35421399

[33] Anliker M Drees D Loacker L Hafner S Griesmacher A Hoermann G. Upregulation of checkpoint ligand programmed death-ligand 1 in patients with paroxysmal nocturnal hemoglobinuria explained by proximal complement activation. J Immunol. (2022) 208:1248–58. doi: 10.4049/jimmunol.2100031, PMID: 35173033

[34] Shao F Gao Y Wang W He H Xiao L Geng X. Silencing EGFR-upregulated expression of CD55 and CD59 activates the complement system and sensitizes lung cancer to checkpoint blockade. Nat Cancer. (2022) 3:1192–210. doi: 10.1038/s43018-022-00444-4, PMID: 36271172

