# Supplementary material for: Changes in serum TIM-3 and complement C3 expression in workers due to Mn exposure
Source: Front Public Health. 2023 Nov 9;11:1289838. doi: 10.3389/fpubh.2023.1289838 (PMC10666638; doi:10.3389/fpubh.2023.1289838)
Supplement: Supplementary file 1 [file Data_Sheet_1.pdf]

Supplementary table 1

Table 1 Concentrations of manganese and its inorganic compounds in the occupational environment（mg/m^3）

Shopfloor	A Post	C-TWA	PC-TWA	peak concentration	3 x PC-TWA
Welding	Weld	0.001-0.127	0.150	0.010-0.292	0.450
Contracting	Weld	0.056-0.142	0.150	0.151-0.378	0.450
Chassis	Weld	0.065-0.131	0.150	0.029-0.210	0.450
Manufacturing	Weld	0.123-0.131	0.150	0.125-0.152	0.450
Trial Production	Weld	0.013-0.113	0.150	0.011-0.182	0.450
Process Department	Weld	0.125	0.150	0.036-0.200	0.450

Supplementary table 2

Table 2 Association of demographic characteristics with WBCs and RBCs

Variables	WBCs(10^9/L)					RBCs(10^{12}/L)				
	Low Mn group		High Mn group		F（P）[a]	Low Mn group		High Mn group		F（P）[a]
	n	$\bar{x}$±s	n	$\bar{x}$±s		n	$\bar{x}$±s	n	$\bar{x}$±s	
Age (years)										
≤8	41	5.77±1.03	41	6.93±1.30	20.299 (<0.001)	41	5.26±0.43	41	5.24±0.36	0.239 (0.626)
>8	31	5.98±0.72	31	7.12±1.35	19.030 (<0.001)	31	5.22±0.31	31	5.27±0.34	0.304 (0.584)
F（P）[b]		0.407(0.526)		0.243(0.623)			0.182(0.671)		0.348(0.557)	
Current smoking										
Yes	28	6.03±0.67	24	7.67±1.53	26.617 (<0.001)	28	5.23±0.34	24	5.22±0.39	0.024 (0.877)
No	44	5.75±1.03	48	6.69±1.07	16.488 (<0.001)	44	5.24±0.41	48	5.26±0.33	0.000 (0.995)
F（P）[b]		1.179(0.281)		7.127(0.010)			0.009(0.926)		0.168(0.684)	
Drinking history										
Yes	5	6.16±0.52	6	8.64±1.84	6.830 (0.040)	5	5.06±0.20	6	5.22±0.48	0.198 (0.672)
No	67	5.83±0.93	66	6.87±1.17	32.279 (<0.001)	67	5.25±0.39	66	5.25±0.34	0.062 (0.803)
F（P）[b]		0.273(0.603)		8.046(0.006)			1.228(0.272)		0.191(0.663)	
BMI										
18.5-23.9	35	5.70±0.91	23	6.85±1.23	18.481 (<0.001)	35	5.20±0.40	23	5.08±0.31	1.386 (0.245)
24.0-27.9	24	5.75±0.95	37	7.08±1.28	22.755 (<0.001)	24	5.21±0.34	37	5.31±0.36	1.083 (0.303)
≥28.0	13	6.36±0.64	12	7.10±1.64	1.540 (0.229)	13	5.38±0.42	12	5.39±0.27	0.005 (0.945)
F（P）[b]		2.875(0.043)		0.601(0.551)			0.770(0.515)		4.445(0.015)	

[a] denotes the difference between the low and high Mn groups for each indicator after stratification of the different variables; [b] denotes the difference between the low and high Mn groups within the low and high Mn groups for each indicator after stratification of the variables.

Supplementary table 3

Table 3 Association of demographic characteristics with Hb and PLT

Variables	Hb(g/L)					PLT (10^9/L)				
	Low Mn group		High Mn group		F（P）[a]	Low Mn group		High Mn group		F（P）[a]
	n	$\bar{x}\pm s$	n	$\bar{x}\pm s$		n	$\bar{x}\pm s$	n	$\bar{x}\pm s$	
Age (years)										
≤8	41	152.83±12.44	41	154.22±9.25	0.224 (0.637)	41	222.01±47.78	41	244.17±49.74	3.837 (0.054)
>8	31	154.00±8.34	31	153.74±8.51	0.118 (0.732)	31	217.01±48.94	31	242.59±47.36	5.184 (0.027)
F（P）[b]		0.492(0.485)		0.127(0.723)			0.441(0.509)		0.225(0.637)	
Current smoking										
Yes	28	151.18±12.99	24	154.33±10.95	0.828 (0.367)	28	226.76±49.12	24	259.03±45.22	3.596 (0.061)
No	44	154.70±9.06	48	153.85±7.77	0.497 (0.483)	44	215.46±47.32	48	235.72±48.51	5.061 (0.029)
F（P）[b]		1.944(0.168)		0.031(0.861)			1.414(0.239)		3.717(0.058)	
Drinking history										
Yes	5	151.60±7.89	6	156.17±9.97	0.181 (0.686)	5	203.28±35.83	6	254.05±39.71	9.343 (0.022)
No	67	153.46±11.03	66	153.82±8.84	0.002 (0.965)	67	221.10±48.78	66	242.53±49.25	6.037 (0.015)
F（P）[b]		0.069(0.794)		0.275(0.602)			0.904(0.345)		0.035(0.852)	
BMI										
18.5-23.9	35	153.56±9.75	23	151.87±7.82	0.418 (0.521)	35	210.96±48.89	23	231.45±39.76	2.667 (0.108)
24.0-27.9	24	152.38±11.64	37	154.19±8.73	0.293 (0.590)	24	229.64±49.55	37	251.73±56.23	3.209 (0.079)
≥28.0	13	154.38±12.93	12	157.58±10.68	0.447 (0.511)	13	228.98±40.47	12	241.15±32.71	0.384 (0.542)
F（P）[b]		0.073(0.974)		1.509(0.229)			1.250(0.299)		1.686(0.193)	

[a] indicates differences in indicators between the low and high Mn groups after stratification of the different variables; [b] indicates differences in indicators within the low and high Mn groups after stratification of the variables.

Supplementary table 4

Table 4 Association of demographic characteristics with TIM-3 and complement C3

Variables	TIM-3(pg/mL)					补体 C3(mg/mL)				
	Low Mn group		High Mn group		χ^2（*P*）[a]	Low Mn group		High Mn group		χ^2（*P*）[a]
	n	*M(P25, P75)*	n	*M(P25, P75)*		n	*M(P25, P75)*	n	*M(P25, P75)*	
Age (years)										
≤8	41	64.68 (56.95,86.85)	41	80.34 (69.99,106.40)	8.489 (0.004)	41	1.07 (0.82,1.26)	41	0.92 (0.64,1.20)	4.421 (0.036)
>8	31	62.30 (51.40,73.04)	31	80.34 (63.75,98.25)	12.385 (<0.001)	31	1.12 (0.98,1.29)	31	0.85 (0.60,1.22)	14.678 (<0.001)
χ^2（*P*）[b]		3.592(0.058)		0.532(0.466)			5.452(0.020)		0.942(0.332)	
Current smoking										
Yes	28	62.36 (51.92,73.91)	24	70.97 (65.45,98.17)	4.785 (0.029)	28	1.04 (0.82,1.26)	24	1.00 (0.62,1.23)	0.947 (0.331)
No	44	65.25 (56.69,81.00)	48	82.12 (66.27,102.28)	13.626 (<0.001)	44	1.14 (1.00,1.29)	48	0.88 (0.63,1.18)	19.933 (<0.001)
χ^2（*P*）[b]		0.242(0.623)		0.797(0.372)			5.825(0.016)		0.154(0.695)	
Drinking history										
Yes	5	62.89 (56.16,96.03)	6	81.68 (66.67,96.47)	1.756 (0.185)	5	1.05 (0.63,1.16)	6	1.22 (0.61,1.40)	0.289 (0.591)
No	67	62.63 (53.82,77.50)	66	79.91 (65.21,103.03)	17.008 (<0.001)	67	1.10 (0.96,1.29)	66	0.88 (0.63,1.17)	19.866 (<0.001)
χ^2（*P*）[b]		0.366(0.545)		0.025(0.876)			2.741(0.098)		1.854(0.173)	χ^2（*P*）[b]
BMI										
18.5-23.9	35	62.53 (51.05,86.83)	23	85.89 (62.55,110.03)	6.226 (0.013)	35	1.13 (0.98,1.30)	23	0.77 (0.62,1.10)	16.862 (<0.001)
24.0-27.9	24	63.78 (57.21,72.35)	37	78.16 (66.31,96.93)	10.789 (0.001)	24	1.05 (0.89,1.28)	37	0.91 (0.62,1.22)	4.777 (0.029)
≥28.0	13	62.34 (53.99,89.75)	12	81.00 (67.68,108.53)	5.434 (0.020)	13	1.09 (0.94,1.27)	12	1.12 (0.84,1.27)	0.508 (0.476)
χ^2（*P*）[b]		0.573(0.902)		0.703(0.704)			5.426(0.143)		3.734(0.155)	

[a] indicates differences in indicators between the low and high Mn groups after stratification of the different variables; [b] indicates differences in indicators within the low and high Mn groups after stratification of the variables.

Supplementary table 5

Table 5 Analytical model of the mediating effect of complement C3 between RBC Mn and TIM-3

Equation	Dependent variable	Independent variable	*B*	R^2	*F*	*p*
Path c	TIM-3	RBC Mn	0.267	0.102	3.132	0.01
Path a	C3	RBC Mn	−0.339	0.134	4.255	0.001
Path b	TIM-3	C3	−0.183	0.131	3.434	0.003
Path c’		RBC Mn	0.205			
